# Stereotactic body radiation therapy for the treatment of localized prostate cancer in men with underlying inflammatory bowel disease

**DOI:** 10.1186/s13014-021-01850-1

**Published:** 2021-07-09

**Authors:** Jonathan W. Lischalk, Seth Blacksburg, Christopher Mendez, Michael Repka, Astrid Sanchez, Todd Carpenter, Matthew Witten, Jules E. Garbus, Andrew Evans, Sean P. Collins, Aaron Katz, Jonathan Haas

**Affiliations:** 1 Department of Radiation Oncology, NYCyberKnife at Perlmutter Cancer Center – Manhattan, Perlmutter Cancer Center at New York University Langone Hospital – Long Island, 150 Amsterdam Ave, New York, NY 11501 USA; 2 Department of Radiation Medicine, Lenox Hill Hospital – Northwell Health, New York, NY 10075 USA; 3 Department of Surgery, New York University Long Island School of Medicine, Mineola, NY 11501 USA; 4 Department of Radiation Oncology, New York University School of Medicine, New York, NY USA; 5 Department of Radiation Medicine, Medstar Georgetown University Hospital, Washington, DC 20007 USA; 6 Department of Urology, New York University Long Island School of Medicine, Mineola, NY 11501 USA

**Keywords:** Prostate, Stereotactic body radiation therapy, Inflammatory bowel disease, Ulcerative colitis, Crohn’s disease

## Abstract

**Background:**

Historically, IBD has been thought to increase the underlying risk of radiation related toxicity in the treatment of prostate cancer. In the modern era, contemporary radiation planning and delivery may mitigate radiation-related toxicity in this theoretically high-risk cohort. This is the first manuscript to report clinical outcomes for men diagnosed with prostate cancer and underlying IBD curatively treated with stereotactic body radiation therapy (SBRT).

**Methods:**

A large institutional database of patients (n = 4245) treated with SBRT for adenocarcinoma of the prostate was interrogated to identify patients who were diagnosed with underlying IBD prior to treatment. All patients were treated with SBRT over five treatment fractions using a robotic radiosurgical platform and fiducial tracking. Baseline IBD characteristics including IBD subtype, pre-SBRT IBD medications, and EPIC bowel questionnaires were reviewed for the IBD cohort. Acute and late toxicity was evaluated using the CTCAE version 5.0.

**Results:**

A total of 31 patients were identified who had underlying IBD prior to SBRT for the curative treatment of prostate cancer. The majority (n = 18) were diagnosed with ulcerative colitis and were being treated with local steroid suppositories for IBD. No biochemical relapses were observed in the IBD cohort with early follow up. High-grade acute and late toxicities were rare (n = 1, grade 3 proctitis) with a median time to any GI toxicity of 22 months. Hemorrhoidal flare was the most common low-grade toxicity observed (n = 3).

**Conclusion:**

To date, this is one of the largest groups of patients with IBD treated safely and effectively with radiation for prostate cancer and the only review of patients treated with SBRT. Caution is warranted when delivering therapeutic radiation to patients with IBD, however modern radiation techniques appear to have mitigated the risk of GI side effects.

## Background

Inflammatory bowel disease (IBD) is a chronic idiopathic inflammatory disorder that affects over one million individuals in the United States and is increasing in incidence [1, 2]. The illness is separated into two distinct subtypes: (1) Crohn’s disease (CD), and (2) ulcerative colitis (UC). Each type carries discrete anatomical and pathophysiological characteristics, but both can fundamentally worsen baseline bowel function [3]. Moreover, IBD carries an increased risk of secondary malignancies [4]. As such, notable efforts have been made to minimize exposure of patients diagnosed with IBD to ionizing radiation even those used for diagnostic purposes [5–8].

Autoimmune disorders including IBD are thought to lead to synergistic increases in toxicity when combined with therapeutic radiation, and great caution has been historically exercised with their combination. A systemic review of the literature identified only eight trials with a total of 144 patients diagnosed with underlying IBD and treated with pelvic radiotherapy [9]. Small case studies have warned against the use of radiation in cases of underlying IBD, and have labeled therapeutic radiation as a “relative contraindication” [10, 11]. Older data from Massachusetts General Hospital (MGH) reported nearly a 50% rate of “severe toxicity” in 28 patients identified from 1970 to 1999 who were treated with abdominopelvic radiotherapy for a variety of malignancies including seven patients with prostate cancer [12]. Such late toxicity included 29% of patients requiring surgical intervention or hospitalization. Therefore, in cases such as prostate cancer where definitive treatment options exist outside of radiation, such alternative local therapies have thus been prudently advocated [12]. It is important to note that anti-inflammatories used to manage IBD can also increase the risk of wound dehiscence and infection following surgery, thus their peri-operative discontinuation may in and of itself flair underlying IBD [13].

In the current era, the toxicity risk with radiation appears to be lower if patients are carefully selected and new radiation modalities are utilized [14–19]. Stereotactic body radiation therapy (SBRT) has been demonstrated to be clinically effective and has become a ubiquitous option for selected men with localized adenocarcinoma of the prostate [20]. However, no literature exists regarding the acute and late toxicity for men with underlying IBD treated with SBRT for prostate cancer. It is reasonable to hypothesize that SBRT using contemporary imaging, advanced treatment planning, and precise radiation delivery could mitigate radiation-related toxicity in this theoretically higher risk cohort.

In this manuscript, we describe our large institutional experience, spanning nearly a decade, and review the clinical outcomes of patients diagnosed with underlying IBD and subsequently treated with SBRT for localized prostate cancer.

## Methods

### Patient eligibility

This single institutional review of patients treated with SBRT for prostate cancer was approved by the local Institutional Review Board (Study # 00001269). All patients were evaluated by a radiation oncologist and deemed appropriate for definitive SBRT. All patients underwent pre-treatment diagnostic tests including clinical examination, PSA, and transrectal ultrasound-guided biopsy. Patients were categorized into D’Amico risk group classifications. All patients underwent placement of fiducial markers in the prostate approximately one week prior to simulation. Fiducial markers were utilized for inter- and intra-fractional image guidance using a robotic radiosurgical platform. Patient IBD history was reviewed in detail to determine the diagnosed subtype of disease as UC, CD, or IBD not otherwise specified. Pre-SBRT IBD treatment (e.g. suppositories, systemic steroids, etc.) was reviewed and documented (Table 2).

### Simulation, planning, and treatment delivery

All patients underwent computed tomography (CT)-based radiation treatment planning simulation (GE Optima 580). An MRI of the prostate was also obtained in the majority of cases at the time of simulation and fused with the primary simulation CT scan at the level of the fiducials to assist in target volume delineation with particular attention paid to the prostate and rectal interface. Patients were recommended enema usage prior to simulation and delivery of each treatment fraction. Target volume contours were generated using previously defined definitions. Nodal radiation was incorporated for those patients deemed to be at high risk for nodal involvement. Organs at risk (OAR) were contoured and included rectosigmoid, bladder, penile bulb, small bowel, and femoral heads.

Clinical target volume included the entire prostate and proximal seminal vesicles. A 5 mm isometric expansion of the CTV was created with a tighter, 3 mm, posterior margin to form the PTV. Dose calculations and planning optimization were performed using Accuray MultiPlan software. Beam angles were created to optimize target volume coverage and minimize exposure of normal structures, with particular attention paid to the rectum. Dosimetric constraints for the aforementioned normal structures were utilized based on institutional standards. All patients were treated using SBRT delivered over five treatment fractions. Treatments were delivered using a robotic radiosurgical platform with prostate motion accounted for in the x-, y-, and z-plane. All patients received intra-rectal amifostine in effort to reduce the risk of radiation-related GI toxicity. Finally, a small number of patients underwent pre-treatment rectal spacer placement, though the majority of patients were treated in an era prior to widespread spacer use.

### Follow-up

Acute toxicity was defined as that occurring within 90 days of treatment completion. Late toxicity was defined as that occurring greater than 90 days after radiotherapy completion. Toxicity was reported using the Common Terminology Criteria for Adverse Events (CTCAE) version 5.0. Patients were followed using serial PSA and clinical examination commonly at 3-month intervals for the first year and subsequently every 6–12 months thereafter. Toxicity was measured from completion of SBRT. Patients who underwent EPIC questionnaires pre and post SBRT were reviewed with a specific focus on bowel habits.

### Statistical analysis

Statistical analysis was performed using the Statistical Package for Social Sciences (SPSS) version 24 (Armonk, NY). The IBD and non-IBD cohort demographic, cancer, and treatment data were compared using Chi-Square analysis.

## Results

### Patient and tumor characteristics

In this single institutional retrospective review, we identified 4,245 patients who were treated with definitive SBRT for localized prostate cancer from 2012 to 2020. Of this cohort, 31 patients (1%) were found to have an underlying diagnosis of IBD prior to undergoing SBRT. The majority of patients in the IBD cohort were between the ages of 60 and 70 years, and there was no significant difference (*p* = 0.58) in the age distribution between the IBD and non-IBD patients when analyzed as a continuous variable. The majority of IBD patients had an excellent documented ECOG performance status of 1 (n = 18, 58%). Androgen deprivation therapy was utilized as a component of treatment in the minority of patients (n = 7, 23%), and was not significantly different relative to the non-IBD cohort.

Within the IBD cohort, the prostate cancer risk grouping was as follows: low (n = 8, 26%), intermediate (n = 17, 55%), and high (n = 6, 19%). Pre-treatment PSA was < 10 ng/mL in the majority of patients (n = 24, 77%). There were no patients found to have locally advanced (i.e. clinical stage T3–4) disease and nearly half were diagnosed with pathologic grade group 2 cancer (n = 13, 42%). There was no statistically significant difference identified between the IBD and non-IBD cohorts from a PSA (*p* = 0.09), clinical stage (*p* = 0.65), pathological grade group (*p* = 0.83), or overall risk group standpoint (*p* = 0.97). Patient and tumor characteristics are listed in Table 1.

**Table 1:** Patient tumor and characteristics

	IBD		No IBD		*p* value
	n	%	n	%	
Age					
< 60 years	4	13%	860	20%	0.58
60–70 years	15	48%	1840	44%	
> 70 years	12	39%	1514	36%	
ECOG					
0	18	58%			
1	2	7%			
No score	11	35%			
PSA (mg/mL)					
< 10	24	77%	3281	78%	0.09
10–20	3	10%	725	17%	
> 20	4	13%	208	5%	
AJCC 8-edition stage					
T1	24	77%	3436	82%	0.65
T2	7	23%	733	17%	
T3–T4	0	0%	45	1%	
Grade group					
1	9	29%	1314	31%	0.83
2	13	42%	1436	34%	
3	4	13%	833	20%	
4	3	10%	418	10%	
5	2	6%	213	5%	
Risk group					
Low	8	26%	1066	25%	0.97
Intermediate	17	55%	2392	57%	
High	6	19%	756	18%	
ADT					
Yes	7	23%	943	22%	0.98
No	24	77%	3271	78%	

### Inflammatory bowel disease diagnosis and severity prior to treatment

Inflammatory bowel disease subtype was most commonly UC (n = 18, 58%) followed by CD (n = 11, 26%) with the remainder of patients having IBD not otherwise specified (n = 2, 16%). The majority of patients (n = 24, 77%) with IBD received medical treatment prior to undergoing radiotherapy. The majority of patients received treatment with local steroidal suppositories (n = 15, 48%). However, systemic treatment was prescribed in nine patients (29%) and included prednisone, hydroxychloroquine, and methotrexate amongst other medications. Anticoagulation use was not common in the IBD cohort (n = 5, 16%). Nearly half of patients (n = 14) within the IBD cohort completed EPIC questionnaires prior to SBRT, which were interrogated for HRQOL bowel domain summary and bowel subscales. Overall, pretreatment HRQOL bowel domain summary scores for the IBD cohort were good with a median score of 90.18 (range, 37.50–100.0). The median pre-SBRT domain-specific HRQOL subscales for bowel function and bother were 94.64 (range, 46.43–100.00) and 91.07 (range, 28.57–100.00), respectively. Inflammatory bowel disease and EPIC questionnaire data details are shown in Tables 2 and 3, respectively.

**Table 2:** Inflammatory bowel disease details

*IBD subtype*	*n*	*%*
Ulcerative colitis	18	58
Crohn’s disease	11	36
Not otherwise specified	2	6
*Pre-SBRT IBD medication*		
Mesalamine	11	36
Sulphasalazine	4	13
Prednisone	2	6
Hydroxychloroquine	1	3
Other^a^	6	19
None	7	23
*Blood thinner use*		
Coumadin	3	10
Aspirin	2	6

*Azathioprine, balsalazide, budesonide, methotrexate, solasodine

**Table 3:** EPIC questionnaire

	Baseline	1 month	3–4 months	6–9 months
	(n = 14)	(n = 5)	(n = 4)	(n = 4)
HRQOL Bowel domain summary				
Median	90	71	64	82
Mean	84	59	62	75
Range	38–100	7–95	23–96	41–96
Domain-specific HRQOL subscales				
Function				
Median	95	71	68	80
Mean	87	59	63	77
Range	46–100	11–89	21–96	50–96
Bother				
Median	91	71	61	84
Mean	81	59	61	74
Range	29–100	4–100	25–96	32–96

### Treatment and dosimetric characteristics

All patients were treated with SBRT to the prostate and proximal seminal vesicles. However, two patients within the IBD group received supplemental pelvic nodal irradiation followed by a prostate and seminal vesicle SBRT boost due to their high-risk disease. The majority of patients were treated to a total dose of 3500 cGy in five fractions (n = 26, 84%). Of the remaining patients, three were treated to 3625 cGy and two patients received a prostate and seminal vesicle boost (2100 cGy and 1950 cGy each in 3 fractions) after nodal irradiation to 4500 cGy in 25 fractions. Most frequently, treatments were delivered on a consecutive day schedule (n = 29, 94%). Only two patients within the IBD group underwent pre-treatment polyethylene glycol gel spacer placement. The remaining patients were treated with amifostine as a rectal protectant.

Dosimetric analysis of the IBD cohort (sans pelvic lymph nodes) was performed with particular attention paid to rectal dosimetry for the majority of patients (n = 27). Target volume coverage in the IBD cohort was excellent with CTV and PTV prescription coverage of 100% and 97%, respectively. Median prescription isodose line was 84% (range, 83–87.5%). Median maximum point dose to the rectum was 3754 cGy with a median rectal V3600 cGy of 0.29 cc. The remaining radiation treatment and dosimetric details are listed in Table 4.

**Table 4:** Treatment and dosimetry results

	IBD		No IBD		*p* value
	n	%	n	%	
Radiation dose					
3500 cGy in 5 fractions	26	84	3139	75	0.46
3625 cGy in 5 fractions	3	10	537	13	
Whole pelvis					
2100 cGy in 3 fractions	1	3	422	10	
1950 cGy in 3 fractions	1	3	86	2	

IBD Dosimetric parameters	Mean	Median	Range		
Target coverage					
CTV VRx (%)	99	100	99–100		
PTV VRx (%)	97	97	95–100		
IDL (%)	84	84	83–88		
Max dose (cGy)	4200	4200	4118–4217		
Rectum Max (cGy)	3800	3800	3604–3919		
Rectum V37.8 (%)	0.1	0	0–0.3		
V37.8 cc (cc)	0.04	0	0–0.2		
Rectum V33.9 (%)	5.2	4.7	1–13		
V33.9 cc (cc)	2.7	2	1–9		
Rectum V29 (%)	15	15	6–25		
V29 cc (cc)	7.7	6.3	3–18		
Rectum V19.2 (%)	37	37	20–56		
V19.2 cc (cc)	19	16	9–34		
Rectum V36 (cc)	0.35	0.29	0.01–1		
Large Bowel Max (cGy)	2600	2600	1239–3689		
Small Bowel Max (cGy)	1100	1100	956–1224		

### Oncologic and toxicity outcomes

Overall, excellent short-term oncologic outcomes were observed regardless of prostate cancer risk group classification in those men diagnosed with underlying IBD. With a median follow up of 22 months, no patients within the IBD cohort were found to have a Phoenix definition biochemical failure. The median and mean PSA nadir for those with IBD was found to be 0.35 ng/mL and 0.76 ng/mL, respectively.

Overall, high-grade acute and late gastrointestinal toxicity was extremely rare (n = 1). Median time to any GI toxicity following SBRT was 22-months. One patient developed grade 3 proctitis requiring hospital management less than 1 month following SBRT. Two additional patients developed grade 2 proctitis requiring outpatient medical management at 22- and 29-months. Hemorrhoids, including hemorrhoidal hemorrhage, were the remaining observed toxicities (n = 3). Toxicity details are illustrated in Table 5. A very small number of patients had EPIC questionnaires available for interrogation following SBRT. Although difficult to generalize given the small patient numbers, bowel quality of life appears to decline 1 month following SBRT with gradual improvements seen at 3–4 months and 6–9 months (Table 3).

**Table 5:** Gastrointestinal toxicity (CTCAE version 5.0)

Toxicity	Time to toxicity (months)	IBD subtype	Risk group	Total dose (cGy)	IDL (%)	Rectal D_max_ (cGy)	Pre-SBRT IBD meds
Grade 3 proctitis	< 1	UC	Int	3500	84	3656	Mesalamine
Grade 2 proctitis	29	UC	Low	3500	85	3852	Prednisone
Grade 2 proctitis	22	UC	Int	3500	83	3860	Mesalamine
Grade 2 hemorrhoids	10	CD	High	3500	86	3839	Methotrexate
Grade 1 rectal hemorrhage	< 1	CD	High	3500	86	3839	Methotrexate
Grade 1 hemorrhoids	22	UC	Int	3500	83	3860	Mesalamine

All patients who developed toxicity were treated to a total dose of 3500 cGy in five fractions. The documented median rectal point dose maximum was 3853 cGy in patients who developed toxicity, which was slightly higher than that of the entire IBD cohort (3754 cGy). Interestingly, the two patients treated with pelvic nodal irradiation were not found to have toxicity. Those patients who underwent pre-treatment rectal spacer placement also did not develop toxicity. Of note, all patients who developed toxicity were receiving IBD medical treatment prior to SBRT often with systemic medication.

## Discussion

The pathophysiological mechanism of radiation-induced damage in IBD remains nebulous. It may be a multifactorial process involving underlying IBD sensitivities to vascular damage, inherently altered DNA repair pathways, and a susceptibility to excessive free radical damage [21–23]. Conversely, radiation has well known immunomodulatory properties and can be notably cytotoxic to immune cells even in low doses yielding anti-inflammatory effects [24–26]. The immunosuppressive effect of radiation has been recently explored for the treatment of COVID-19-related pneumonia with mixed results [27]. Thus, it is fair to ask whether radiation might suppress the dysregulated and hyperactive mucosal immune response in the rectum leading to improved symptomatology, which is intriguingly what was observed by a Gestaut et al. [16].

Gestaut et al. reported a group of 18 patients with exclusively prostate cancer treated with a variety of radiation modalities including three-dimensional conventional radiation therapy (3DCRT), intensity modulated radiation therapy (IMRT), and low dose rate brachytherapy (12 patients received EBRT). The majority of patients (79%) required IBD medication prior to and during radiation treatment. Similar to our study, remarkably low GI toxicity was observed with no instances of grade 3 + toxicity identified. Of note, grade 2 proctitis was observed more frequently following 3DCRT, prompting the authors to advocate for the use of more conformal radiation techniques (e.g. IMRT). The use of “specialized” radiation techniques to minimize late toxicity was also observed in the aforementioned MGH publication [12]. Interestingly, the overall cohort demonstrated decreased grade 1 diarrhea immediately following radiation, and the authors postulate this may be a result of radiation’s effect in mitigating IBD symptoms, at least in the acute setting.

Excess GI toxicity risk in patients with IBD is difficult to compare to a non-IBD cohort due to limited patient numbers. However, Murphy et al. reviewed 16 patients with underlying IBD who were treated with EBRT for prostate cancer and attempted to identify case controls without IBD to delineate a comparative risk profile [17]. Interestingly, no difference in grade 2 + toxicity was identified between patients with IBD and case controls. Late grade 2 toxicity was low at 10% (vs. 17% in Gestaut et al.), which could be explained by the lower numbers of patients who received concurrent IBD medication, (47% versus 79%) perhaps indicative of the lower severity of underlying IBD in the Murphy et al. patient cohort [16].

In a unique publication, Feagins et al. reviewed a cohort entirely of IBD patients who did or did not receive radiation for prostate cancer treatment—distinct from all other publications, which reviewed radiation patients who did or did not have IBD. Veterans Administration data was reviewed from 1996 to 2015 of patients treated for prostate cancer with a radiation versus a non-radiation modality [15]. Baseline characteristics between the radiation and non-radiation cohorts did not appear to be different. However, there was a twofold higher rate of IBD flares within the first year after radiation relative to those patients who did not receive radiotherapy as primary treatment. Nevertheless, there were no differences in high-grade toxicities (i.e. hospitalization or surgeries). The use of “IBD flare” as a metric for toxicity in this trial is unique—only Annede et al. published similarly. Whether IBD flares represent the equivalent of a low-grade CTCAE toxicity is difficult to establish, but highlights the importance of comparing apples to apples across publications with respect to toxicity metrics.

Attempts to exploit the inverse square law to minimize rectal toxicity using I-125 LDR brachytherapy was reported by Pai et al. [28]. A total of 13 patients with IBD received I-125 implants and a markedly elevated rate of grade 3 + toxicity was observed at 23% and 15% for acute and late toxicity, respectively. Importantly, all patients who developed high-grade late toxicity underwent rectal biopsies shortly after brachytherapy implant. Similar high-grade toxicity was observed in patients with IBD treated with EBRT for colorectal cancer in the peri-operative setting [29]. These studies allude to an augmented toxicity risk with surgical manipulation of the pelvis in IBD cases following radiation, and highlight the importance of avoiding elective rectal interventions shortly after radiotherapy regardless of the presence of underlying bowel disease.

Literature for more heterogeneous groups of malignancies have highlighted several additional risk factors associated with toxicity including concurrent chemotherapy use and IBD location. Annede et al. reported a diverse group of patients with pelvic malignancies and IBD (12 of 28 with prostate cancer) treated with radiation from 1989 to 2015 [30]. External beam radiotherapy (primarily older 2D techniques) was delivered to a median dose of 53 Gy. Of note, no patients had “active IBD” at baseline in this study. Only rectal IBD anatomical location was significantly correlated with IBD exacerbation within 6 months after radiation. With a median follow up of nearly 6 years, grade 3 + GI toxicity rate was 11% and 4% for acute and late toxicity, respectively. Johns Hopkins Hospital reported on 24 patients with IBD who underwent primarily chemoradiation with conventional techniques for a heterogeneous group of malignancies (only one prostate cancer). High-grade toxicity (3 +) was reported at 21% and 8% for acute and late toxicity, respectively, with concurrent chemotherapy being the primary driver of toxicity.

Many of the aforementioned patients were treated with antiquated radiation techniques in an era when 3D planning did not exist and modern image guided radiation therapy (IGRT) had yet to be developed. Systemic review of the literature in this setting of a heterogeneous cancer cohort and EBRT treatment technique estimated the mean rate of acute and late grade 3 + GI toxicity to be quite high at 20% (range: 11–27%) and 15% (range: 0–23%), respectively [9]. Contemporary radiation incorporates detailed imaging including prostate MRI, exquisitely conformal radiation planning, and inter- and intra-fractional IGRT. Such improvements have led to decreases in radiation toxicity without a detriment in oncologic outcome in the non-IBD setting [31]. The most notable modern advancement from a rectal toxicity standpoint is the placement of a polyethylene glycol-based gel that creates an artificial space between the posterior aspect of the prostate and anterior aspect of the rectum. Placement of hydrogel spacers has consistently demonstrated superior dosimetry across nearly all dose volume parameters, and has translated into low rates of GI toxicity and excellent patient reported quality of life outcomes [32–37]. Advantages with the utilization of rectal spacers have been demonstrated not only in conventional radiotherapy treatments, but also in SBRT delivered with advanced MRI-guided radiotherapy. Alongi et al. report improvements not only in rectal dose sparing but also target volume coverage [38]. Furthermore, Cuccia et al. demonstrated rectal spacers seemed to “stabilize” the prostate by minimizing rotational antero-posterior shifts during MRI-guided radiotherapy delivery [39]. For patients with underlying IBD, such dramatic improvements in rectal dose and toxicity may be a panacea. In our particular IBD cohort, two patients underwent rectal spacer placement, and neither developed gastrointestinal toxicity.

Limitations of the present study include its retrospective nature, relatively short median follow up, and limited patient numbers, which is a consequence of the rarity of the clinical situation. Nevertheless, this is one of the largest cohorts ever reported of patients with IBD treated with modern radiation, the most detailed and homogenous from a radiation technique standpoint, and the only report detailing outcomes following SBRT. Caution is warranted and careful selection of patients appears to be crucial. If possible, treatment should be avoided during active IBD flares, particularly if localized to the rectum, and patients should be well managed medically prior to treatment. Strong consideration should be made for use of rectal spacers in effort to minimize rectal exposure to radiation. Conformal radiation with rigorous IGRT should be utilized and following radiation elective biopsies of the rectum should be avoided at all costs. Nevertheless, contrary to historical lore, prostate radiotherapy in patients with underlying IBD does not appear to be as toxic in the modern era.

## Conclusion

Caution is warranted when delivering therapeutic radiation to patients with IBD; however, modern radiation techniques appear to have mitigated the risk of GI side effects. To date, this is one of the largest groups of patients with IBD treated with radiation for prostate cancer and the only review of patients treated with SBRT. Delivery of 5-fraction SBRT using a non-coplanar robotic platform with tight posterior margins result in low rates of gastrointestinal toxicity with no significant detriment on oncologic outcome.

## Data Availability

The datasets used and/or analyzed during the current study are available from the corresponding author on reasonable request.

## Abbreviations

IBD: Inflammatory bowel disease
CD: Crohn’s disease
UC: Ulcerative colitis
MGH: Massachusetts General Hospital
SBRT: Stereotactic body radiation therapy
PSA: Prostate specific antigen
CT: Computed tomography
MRI: Magnetic resonance imaging
OAR: Organs at risk
CTV: Clinical target volume
PTV: Planning target volume
GI: Gastrointestinal
CTCAE: Common Terminology Criteria for Adverse Events
EPIC: The Expanded Prostate Cancer Index Composite
SPSS: Statistical Package for Social Sciences
ECOG: Eastern Cooperative Oncology Group
ADT: Androgen deprivation therapy
HRQOL: Health Related Quality of Life
cGy: Centigray
DNA: Deoxyribonucleic acid
3DCRT: Three-dimensional conventional radiation therapy
IMRT: Intensity modulated radiation therapy
EBRT: External beam radiation therapy
LDR: Low dose rate
IGRT: Image guided radiation therapy
